# Highly prevalence of vitamin D deficiency among Brazilian women of reproductive age

**DOI:** 10.1590/2359-3997000000216

**Published:** 2016-09-26

**Authors:** Vinicius Medina Lopes, Joaquim Roberto Costa Lopes, Jean Pierre Barguil Brasileiro, Ingrid de Oliveira, Ricardo Peclat Lacerda, Marcos Renato Dib Andrade, Natália Ivet Zavattiero Tierno, Roberto Costa Cavalcante de Souza, Luiz Augusto Casulari Roxo da Motta

**Affiliations:** 1 Departamento de Reprodução Humana Instituto de Vídeo Endoscopia e Reprodução Humana Brasília DF Brasil Departamento de Reprodução Humana, Instituto de Vídeo Endoscopia e Reprodução Humana (VERHUM), Brasília, DF, Brasil; 2 Departamento de Reprodução Humana Centro de Medicina Reprodutiva Salvador BA Brasil Departamento de Reprodução Humana, Centro de Medicina Reprodutiva (Cenafert), Salvador, BA, Brasil; 3 Departamento de Embriologia Instituto de Vídeo Endoscopia e Reprodução Humana Brasília DF Brasil Departamento de Embriologia, Instituto de Vídeo Endoscopia e Reprodução Humana (VERHUM), Brasília, DF, Brasil; 4 Departamento de Endocrinologia Hospital Universitário de Brasília Brasília DF Brasil Departamento de Endocrinologia, Hospital Universitário de Brasília (HUB), Brasília, DF, Brasil

**Keywords:** Vitamin D, infertility, prevalence, vitamin D deficiency, polycystic ovary syndrome

## Abstract

**Objective:**

Vitamin D has several metabolic functions and possible reproductive functions. This study aimed to determine the prevalence of vitamin D deficiency among Brazilian women of reproductive age, and to evaluate the relationship between serum 25-hydroxyvitamin D [25(OH)D] concentrations and infertility causes.

**Subjects and methods:**

This retrospective cross-sectional study evaluated data from a private Brazilian assisted reproduction center that were collected between January 1 and May 5, 2012. Serum 25(OH)D concentrations were measured and compared for infertile and fertile women. Concentrations of 25(OH)D that were < 20 ng/mL were defined as deficiency and concentrations of 21-30 ng/mL were defined as hypovitaminosis D.

**Results:**

Among the 369 evaluated women, 81.1% exhibited hypovitaminosis D and 32.0% exhibited deficiency. The infertile and control patients did not exhibit any significant differences in the prevalence of vitamin D deficiency (30.2% vs. 35%, respectively; p = 0.33) or in the mean 25(OH)D concentrations (24.3 ± 7.9 ng/mL vs. 23.8 ± 8.7 ng/mL, respectively; p = 0.51). Furthermore, there were no significant differences in the mean 25(OH)D concentrations among subgroups of patients with single infertility factors, or between these subgroups and the control group.

**Conclusions:**

A high proportion of Brazilian women of reproductive age exhibited vitamin D deficiency, regardless of their fertility status. Thus, it may be useful to evaluate this population for vitamin D deficiency, although future studies are needed to determine whether this deficiency might affect the outcomes of treatments for infertility.

## INTRODUCTION

Vitamin D is a secosteroid (similar to a steroid, but with a “broken” carbon ring) that plays an important role in several metabolic processes and may influence women’s reproductive physiology ( 1 ). For example, the cervical and uterine tissues, vaginal and cervical epithelia, endometrial and epithelial cells of the fallopian tubes, ovaries, and pituitary glands contain receptors and enzymes that are involved in vitamin D metabolism ( 1 ). Therefore, inadequate serum concentrations of 25(OH)D might be associated with infertility factors, such as chronic anovulation, endometriosis, and even breast cancer ( 2 ). Several studies have found higher pregnancy rates in women with normal 25(OH)D concentrations than in women with low 25(OH)D concentrations ( 3 , 4 ). Furthermore, a review article concluded that vitamin D may be related to the pathogenesis of endometriosis because of its immunomodulatory and anti-inflammatory effects ( 5 ). However, the authors of that review also emphasized the need for further studies to examine the effect of vitamin D concentrations on the development of endometriosis. Several studies have also examined the relationship between vitamin D metabolism and the pathogenesis of polycystic ovary syndrome (PCOS). Among women with PCOS, low serum concentrations of 25(OH)D were associated with obesity, endocrine disorders, and metabolic disorders ( 1 ). Moreover, another study examined the effect of serum concentrations of 25(OH)D on endometrial thickness among 193 patients who were undergoing controlled ovarian hyperstimulation and in vitro fertilization. The blood samples for vitamin D testing were obtained on the day of oocyte retrieval, although there was no significant relationship between endometrial thickness and different serum concentrations of 25(OH)D ( 6 ).

During the last decade, we have witnessed a global epidemic of vitamin D deficiency. One review examined 195 studies of vitamin D status that included > 168,000 people from 44 countries, and found that the prevalence of vitamin D deficiency (< 20 ng/mL) was 37.3% ( 7 ). However, studies investigating vitamin D status in low-risk Brazilian populations are rare ( 8 ). One study found that the average serum 25(OH)D concentration was 31.6 ± 12.4 ng/mL among 99 adults (average age: 67 years) who regularly played outdoor sports ( 9 ). Other authors have reported that hypovitaminosis D was observed in 71.2% of hospitalized seniors and in 43.8% of seniors who were treated as outpatients, and that the women exhibited lower vitamin D concentrations compared to the men in both groups ( 10 ). These findings are troublesome, as vitamin D is very important for bone metabolism and other biological systems. For example, 25(OH)D concentrations exhibit moderate-to-strong inverse associations with cardiovascular diseases, inflammation, reduced cognitive function, glucose metabolism disorders, weight gain, serum lipid concentrations, infectious diseases, reduced physical function, and all-cause mortality ( 11 ). Furthermore, the modern lifestyle is associated with a progressive increase in the prevalence of hypovitaminosis ( 12 ). Therefore, it is important to identify high-risk groups and the regional prevalences of hypovitaminosis D in order to implement effective health policies.

To the best of our knowledge, this is the first study regarding hypovitaminosis D among infertile Brazilian women of child-bearing age, and we have not found any similar studies that examined vitamin D status in the city of Brasilia (in Distrito Federal, the Midwest Region of Brazil). Therefore, this study was designed to determine the prevalence of hypovitaminosis D among women who visited an assisted reproduction clinic in Brasilia. Our secondary objective was to determine whether serum concentrations of vitamin D varied according to fertility status, age, or the cause(s) of infertility.

## SUBJECTS AND METHODS

This study used a retrospective cross-sectional design to examine records from patients who visited a Brazilian assisted reproduction center between January 1, 2012 and May 15, 2012. The study’s design was reviewed and approved by the institutional review boards of the assisted reproduction clinic and Armed Forces Hospital; all procedures were performed in accordance with the 2008 revision of the Declaration of Helsinki.

### Subjects

We included all infertile patients who were treated at the Video Endoscopy and Human Reproduction Center between January 1 and May 15, 2012 (the summer and autumn seasons in Brazil). The control group included 30–45-year-old women who were treated at the clinic, but had not complained of infertility. Both groups underwent vitamin D testing at the same time, and all tests were performed at the same laboratory. We excluded women who were receiving vitamin D supplementation or drugs that could interfere with the absorption, excretion, or serum concentration of vitamin D.

### Variables and definitions

Cases of infertility were defined as couples who failed to conceive after at least 1 year of regular unprotected intercourse. The patients’ electronic health records were used to collect information regarding the infertility’s cause(s), which was based on the patient’s recall, a physical examination, and additional examinations. We also collected data regarding the patients’ age and serum 25(OH)D concentration. Only couples who completed the full infertility evaluation were included in the calculations of the prevalences for each infertility factor (alone or in association with other factors). The infertility factors were defined using the following criteria:

1. Male factors: sperm with an abnormal pattern on at least two occasions, on the basis of the World Health Organization’s criteria ( 13 ).

2. Low ovarian reserve: an antral follicle count of < 11 in both ovaries, which was determined using transvaginal ultrasonography during days 2–5 of the menstrual cycle.

3. PCOS: based on the Rotterdam consensus criteria ( 14 ).

4. Tubal factors: women who presented with tubal obstruction or peritoneal adhesions and a history of tubal ligation, hysterosalpingography, or videolaparoscopy.

5. Endometriosis: women who were diagnosed with endometriosis based on videolaparoscopy and/or at least two vaginal ultrasounds (> 30 days apart), or women who exhibited a debris-containing ovarian cyst that suggested endometrioma.

6. Multiple factors: couples who presented with at least two of the infertility factors that are listed above.

7. Unexplained infertility: couples who did not exhibit any known infertility factors after completing the evaluation.

Vitamin D deficiency was defined as 25(OH)D concentrations of < 20 ng/mL, vitamin D insufficiency was defined as 25(OH)D concentrations of 20–30 ng/mL, and normal vitamin D status was defined as 25(OH)D concentrations of > 30 ng/mL ( 8 ). The 25(OH)D assay was performed using the LIAISON^®^ device and a chemiluminescence kit (DiaSorin, Stillwater, MN), which has a detection range of 4–150 ng/mL, an intra-assay variability of 3.2–8.1%, and an inter-assay variability of 6.9–2.7% ( 15 ).

Unless otherwise stated, we included all women in the analyses, regardless of whether they had completed the infertility evaluation, to evaluate the prevalence of vitamin D deficiency. We also compared the mean serum 25(OH)D concentrations among subgroups of patients who exhibited a single infertility factor after the full infertility evaluation. Furthermore, we compared the serum 25(OH)D concentrations between the control group and the subgroups with a single infertility factor, despite the possibility that cases might present with multiple infertility factors. Moreover, we compared the mean 25(OH)D concentrations of women with and without laparoscopy-confirmed endometriosis.

### Statistical analysis

The chi-square test was used to evaluate the frequency distribution among groups. The non-parametric Kruskal-Wallis test was used to compare the mean serum concentrations of 25(OH)D. Multivariate logistic regression was adjusted for age and was also used to compare the 25(OH)D concentrations between the infertile and control groups; the results were reported as adjusted odds ratios (OR) and their respective 95% confidence intervals (CIs). All statistical analyses were performed using Stata software (StataCorp), and differences were considered statistically significant at a p-value of < 0.05.

## RESULTS

In this study, the 369 eligible women (21–47 years old) were divided into two groups: 232 infertile women and 137 controls. The infertile women exhibited an average age of 35.5 ± 4.6 years, and the control group exhibited an average age of 36.5 ± 4.3 years (p = 0.1). A total of 116 infertile couples (50%) completed the infertility evaluation, and a summary of their infertility factors is shown in Table 1 . The most common single factor was male factors (12.6%), although male factors (29.4%), tubal factors (28.6%), and low ovarian reserve (27.7%) exhibited similar frequencies among couples with multiple factors. Both male and female factors were observed in 35 couples (30.2%), and unexplained infertility was observed in 22.4% of the couples who completed the infertility evaluation.

**Table 1:** Frequency of infertility factors alone or in association with other factors among 116 couples who completed the infertility evaluation

Factors	Alone, n (%)	Associated with others, n (%)
Male	15 (12.6)	35 (30.2)
Tubal	12 (10.1)	33 (28.5)
Low ovarian reserve	11 (9.2)	32 (27.6)
Polycystic ovary syndrome	12 (10.1)	21 (18.1)
Endometriosis	3 (2.5)	11 (9.2)
Unexplained infertility	26 (22.4)	----

Table 2 presents the distributions of the 369 women’s serum 25(OH)D concentrations according to fertility status. Approximately 81.1% of the 369 women exhibited some degree of hypovitaminosis D. The 232 infertile women exhibited a 25(OH)D concentration range of 9.8–55 ng/mL, and 190 women (81.9%) exhibited some degree of hypovitaminosis D. The control group exhibited a 25(OH)D concentration range of 7.7–48 ng/mL, and 79.5% of the women exhibited some degree of hypovitaminosis D. When we compared the infertile and control groups, we found no significant difference in their frequencies of vitamin D deficiency (p = 0.33) or mean 25(OH)D concentrations (24.3 ± 7.9 ng/mL vs. 23.8 ± 8.7 ng/mL, respectively; p = 0.51). There was also no significant difference in the 25(OH)D concentrations of the control and infertile groups after adjustment for age (OR: 0.87; 95% CI: 0.51–1.49).

**Table 2:** Distribution of women according to serum concentrations of 25(OH)D

	< 20 ng/ mL	20–30 ng/mL	> 30 ng/mL
Infertile group (n = 232)	70 (30.2%)	120 (51.7%)	42 (18.1%)
Control group (n = 137)	48 (35%)	61 (44.6%)	28 (20.4%)
Total (n = 369)	118(32%)	181 (49.1%)	70 (18.9%)

Chi-square test, p = 0.41.

Table 3 shows the mean serum 25(OH)D concentrations from the 116 couples who completed the infertility evaluation, according to the single infertility factors; no significant differences were observed. Table 4 shows the relationships between the mean serum 25(OH)D concentrations in the control group and the concentrations in the infertile women according to the infertility factors; no significant differences were observed. The mean 25(OH)D concentrations were not significantly different when we compared the 21 women with endometriosis (25.2 ± 7.9 ng/mL) and the 25 women without endometriosis (25.1 ± 8.5 ng/mL) (p = 0.81).

**Table 3:** Comparing the mean 25(OH)D concentrations (ng/mL) according to infertility factors among couples with single-cause infertility

Infertility factors	Mean ± SD^1^ (n)	Mean ± SD^2^ (n)	P*
Male vs. tubal	26.2 ± 9.2 (15)	22.0 ± 6.2 (12)	0.34
Male vs. low ovarian reserve	26.2 ± 9.2 (15)	23.8 ± 8.6 (12)	0.46
Male vs. ^†^PCOS	26.2 ± 9.2 (15)	23.3 ± 6.4 (13)	0.55
Male vs. unexplained infertility	26.2 ± 9.2 (15)	23.3 ± 8.6 (26)	0.37
Tubal vs. low ovarian reserve	22.0 ± 6.2 (12)	23.8 ± 8.6 (12)	0.73
Tubal vs. ^†^PCOS	22.0 ± 6.2 (12)	23.3 ± 6.4 (13)	0.77
Tubal vs. unexplained infertility	22.0 ± 6.2 (12)	23.3 ± 8.6 (26)	0.83
Low ovarian reserve vs. ^†^PCOS	23.8 ± 8.6 (12)	23.3 ± 6.4 (13)	0.85
Low ovarian reserve vs. unexplained infertility	23.8 ± 8.6 (12)	23.3 ± 8.6 (26)	0.99
^†^PCOS vs. unexplained infertility	23.3 ± 6.4 (13)	23.3 ± 8.6 (26)	0.92

^1 ^The mean 25(OH)D concentrations for women who presented with the first factor each row. ^2 ^The mean 25(OH)D concentration for women who presented with the second factor in each row. * P-values were obtained using the Kruskal-Wallis test. ^† ^PCOS = polycystic ovarian syndrome.

**Table 4:** Comparing the mean 25(OH)D concentrations (ng/mL) from infertile patients who completed the full infertility evaluation and the control group

Factors	Mean ± SD	Cases	P*
Male	26.4 ± 9.2	35	0.15
Tubal	23.9 ± 7.3	33	0.75
Low ovarian reserve	24.7 ± 7.5	32	0.48
Polycystic ovary syndrome	25.2 ± 8.7	21	0.46
Unexplained infertility	23.3 ± 8.6	26	0.83
Endometriosis	27.0 ± 9.4	11	0.27
Control	23.8 ± 8.7	137	-

* The Kruskal-Wallis test was used to compare the results from the infertile patients and the control group.

## DISCUSSION

The present study did not detect a significant difference in the mean serum 25(OH)D concentrations of the infertile and control groups. However, 25(OH)D concentrations in both groups were < 30 ng/mL, which is the lowest level that is recommended by the American and Brazilian Societies of Endocrinology ( 8 , 16 ). Furthermore, we found that the prevalence of vitamin D deficiency was 35.0% among the women of reproductive age in this study. These results are consistent with the prevalence of 36% that has been reported in the American general population ( 17 ). In contrast, a Mongolian study that examined 420 women who were 18–44 years old during the spring found that 415 women (98.8%) exhibited 25(OH)D concentrations of < 20 ng/mL and that only 1 woman (0.2%) exhibited a 25(OH)D concentration of > 30 ng/mL ( 18 ). Brazil’s tropical location favors sun light exposure, which is the most important factor for maintaining vitamin D homeostasis. Therefore, it would be logical to assume that Brazil would have a lower prevalence of hypovitaminosis D compared to subtropical countries. Nevertheless, we found a high prevalence of vitamin D deficiency among Brazilian women. This may be related to the low levels of vitamin D in the Brazilian diet, and the infrequent use of multivitamin supplements ( 19 ). Furthermore, vitamin D deficiency may be related to lifestyle changes that reduce exposure to sunlight and/or the use of sunscreen ( 20 ). Moreover, environmental pollution may be related to vitamin D deficiency, although this factor is not present in Brasilia, and this relationship likely does not explain the high prevalence of vitamin D deficiency in the present study.

The melanin in skin acts as a natural sunscreen by absorbing ultraviolet radiation, and people with darker skin pigmentation may require prolonged sun exposure to synthesize the same amount of vitamin D, compared to people with lighter skin ( 21 ). Ethnicity may also influence 25(OH)D concentrations, as an American study of 2,972 women reproductive age found that the prevalences of vitamin D deficiency were 42.4 ± 3.1% among African descendants and 4.2 ± 0.7% among the Caucasian participants ( 22 ). Furthermore, the prevalence of vitamin D deficiency is 30–90% in the Middle East and North Africa ( 23 ). However, it would be extremely difficult to account for ethnicity in Brazilian studies, as the Brazilian population is descendent from African, indigenous Indian, and European populations. Therefore, we did not consider ethnicity or skin phototype as variables of interest in the present study.

In the present study, 70 infertile women (30.2%) exhibited vitamin D deficiency. Other studies of women who were undergoing in vitro fertilization have found a wide range of vitamin D deficiency rates, such as 21–27% in the US ( 3 , 4 ), 45.9% in Italy ( 24 ), and 98.8% in Iran ( 25 ). However, this wide range is unsurprising, as ethnicity is related to both food intake and the metabolism of vitamin D ( 26 ). In Brazil, a study of 894 adults (mean age: 58.1 ± 12.0 years) in the State of Pernambuco found that the mean 25(OH)D concentration was 26.0 ± 10.3 ng/mL and that the prevalence of vitamin D deficiency was 28.5% ( 27 ). Another Brazilian study of elderly individuals (mean age: 79.1 years) in São Paulo during the winter found that the mean 25(OH)D concentration was 11.6 ng/mL ( 28 ). However, these discrepancies may be related to differences in the patients’ mean age and the season of the analysis. In contrast, the present study was conducted during the summer and autumn seasons, and only examined women of reproductive age, which would suggest that age and seasonality did not influence the serum 25(OH)D concentrations in the infertility and control groups.

We did not identify any significant differences in the 25(OH)D concentrations from the infertility factor subgroups, although these analyses only compared single infertility factors. However, the highest 25(OH)D concentrations were observed in the subgroup that only exhibited male factors (26.84 ± 8.61 ng/mL), and the greatest difference in 25(OH)D concentrations was observed when we compared the groups with male factors (26.2 ± 9.2 ng/mL) and tubal factors (22.0 ± 6.2 ng/mL). Nevertheless, these differences were not statistically significant.

To the best of our knowledge, there are no studies that have evaluated the association of hypovitaminosis D with tubal factors, unexplained infertility, or low ovarian reserve. However, the associations of hypovitaminosis D with PCOS and endometriosis warrant further discussion. For example, the association of low 25(OH)D concentrations with PCOS remains controversial. In the present study, women with PCOS exhibited low 25(OH)D concentrations (25.2 ± 8.7 ng/mL). Similarly, a study of 196 female Iranian students (16–20 years old) found that women with PCOS exhibited lower mean 25(OH)D concentrations, compared to the control group (9.7 ± 4.8 ng/mL vs. 12.3 ± 11.9 ng/mL; p = 0.04) ( 29 ), although these low levels may be associated with the traditional Islamic clothing, which can reduce women’s exposure to sun light. Nevertheless, we did not observe any difference in the 25(OH)D serum concentrations of infertile patients with PCOS or other infertility factors, or in 25(OH)D concentrations of the PCOS and control groups. These results conflict with previous studies’ findings that hypovitaminosis D is associated with PCOS ( 1 ). Furthermore, other authors have suggested that vitamin D deficiency is associated with PCOS only among obese women ( 30 , 31 ). Moreover, another study found that hypovitaminosis D was associated with insulin resistance, regardless of the patient’s body mass index ( 32 ). Unfortunately, we were unable to evaluate the relationships of obesity and/or body mass index with hypovitaminosis D among women with PCOS, as these data were not included in the records that we retrospectively analyzed.

In the present study, 21 infertile women were diagnosed with endometriosis, although only 14 women completed the infertility evaluation and only 3 women did not exhibit other infertility factors. Nevertheless, we did not observe any significant difference in the 25(OH)D concentrations when we compared women who did and did not have a diagnosis of endometriosis. Furthermore, we did not observe any significant difference when we compared women with endometriosis and the control group. These results agree with the findings of Agic and cols. ( 33 ), but conflict with Somigliana and cols.’s findings that women with endometriosis had higher 25(OH)D concentrations, and that the severity of the endometriosis was associated with the 25(OH)D concentrations ( 34 ). Moreover, another study found high concentrations of 1,25(OH)D in women with endometriosis, although there was no significant difference when their concentrations were compared to 25(OH)D concentrations in women without endometriosis ( 35 ). Therefore, our findings cannot definitively resolve the controversy regarding the possible relationship between endometriosis and 25(OH)D concentrations.

In conclusion, the present study revealed that only 18% of the women of reproductive age in Brasilia exhibited normal 25(OH)D serum concentrations, and that 32.9% of the women fulfilled the criterion for vitamin supplementation (serum 25(OH)D concentrations of < 20 ng/mL). Nevertheless, we did not observe any significant difference in 25(OH)D concentrations according to fertility status, which precludes any conclusion regarding an association between infertility and vitamin D status. Therefore, given the controversy regarding the associations of vitamin D status with endometriosis and PCOS, larger prospective studies are needed to provide more definitive data.
